# Prevalence of Human T-Cell Leukemia Virus Type 1 Carrier in Japanese Pregnant Women in 2013

**DOI:** 10.14740/jocmr2097w

**Published:** 2015-04-08

**Authors:** Shunji Suzuki, Masanobu Tanaka, Hideo Matsuda, Yuki Tsukahara, Yasushi Kuribayashi, Akihito Nakai, Ryoichiro Miyazaki, Naoki Kamiya, Akihiko Sekizawa, Nobuko Mizutani, Katsuyuki Kinoshita

**Affiliations:** a Japan Association of Obstetricians and Gynecologists, Japan

## To the Editor

Since September 2010, in Japan serological screening for the detection of human T-cell leukemia virus type 1 (HTLV-1) antibodies can be performed for all women during pregnancy with the Japanese public funds for strategies for prevention of HTLV-1 vertical transmission, because Japan, especially Kyushu area, has been reported to be one of the areas of highest prevalence of HTLV-1 in the world [1, 2]. In our previous study [3], we examined the prevalence of HTLV-1 carrier in Japanese pregnant women according to the implement rate and results of HTLV-1 screening and confirmation tests of women who gave births in Japan in 2011. The total rates of positive HTLV-1 screening tests and positive western blot (WB) test in positive screening tests were 0.32% and 49.8%, respectively in 2011. Considering the response rate and the rate of implementation of WB test, the number of HTLV-1 carrier in Japanese pregnant women in 2011 was estimated to be 1,560 (0.15%). In addition, although the number of delivery in Kyushu area was only 14% of Japanese deliveries, 53% of HTLV-1 carrier of Japanese pregnant women was present in Kyushu area.

Recently, the migration of Japanese people from Kyushu area to the metropolitan areas has been thought to contribute to a significant decrease of HTLV-1 carriers in Kyushu area and an increase in Kanto (including Tokyo) area in Japan [1, 4]. To confirm this migration in Japanese pregnant women, on December 2014, we requested again 2,544 obstetrical facilities that are members of Japan Association of Obstetricians and Gynecologists (JAOG) to provide information of HTLV-1 tests in pregnant women who delivered at ≥ 22 weeks’ gestation in 2013. A total of 1,356 (53.3%) of 2,544 obstetrical facilities responded and information on a total of 538,167 women, accounting for approximately 54% of all deliveries that occurred in Japan during the study period (approximately 1,001,800 births) was provided.

In 2013, the total rates of positive HTLV-1 screening tests and positive WB test in positive screening tests were 0.35% and 50.8%, respectively. Considering the response rate and the rate of implementation of WB test, the number of HTLV-1 carrier in Japanese pregnant women in 2013 was estimated to be 1,780 (0.18%). Table 1 shows the difference in the estimated number of HTLV-1 carrier based on positive WB test by area in Japan between 2011 and 2013. The estimated number of HTLV-1 carrier in 2013 seemed to be more than that in 2011, especially in the northeast and southwest (Kyushu) areas. In addition, 51% of HTLV-1 carrier of Japanese pregnant women was present in Kyushu area, although the number of delivery in Kyushu area was only 13% of Japanese deliveries in 2013.

**Table 1:** Results of HTLV-1 Western Blot Tests and Estimated Number of Human T-Cell Leukemia Virus Type 1 Carrier in Pregnant Women Who Delivered at ≥ 22 Weeks’ Gestation in 2011 and 2013 by Area in Japan

	2011		2013	
	WB positive rate (%)	Estimated HTLV-1 carrier	WB positive rate (%)	Estimated HTLV-1 carrier
Northeast area	0.084	90	0.140	150
Kanto (around Tokyo)	0.062	230	0.067	240
Central area	0.083	120	0.080	110
Kinki (around Osaka)	0.151	240	0.163	270
West central area	0.113	110	0.104	100
Kyushu area (southwest area)	0.596	820	0.663	910
Total	0.154	1,560	0.178	1,780

Values are presented as percentage or number. HTLV-1: human T-cell leukemia virus type 1; WB test: western blot test.

Although the migration of Japanese people from Kyushu area to the metropolitan areas has been supposed to contribute to a significant decrease of HTLV-1 carriers in Kyushu area, the estimated number of HTLV-1 carrier of pregnant women seemed to be increased in Kyushu area. In addition, the estimated rate of HTLV-1 carrier in pregnant women in Kyushu area was still significantly higher than that in the other areas (P < 0.01 by the Chi-square test). Therefore, there are still remaining problems concerning the locality for strategies for prevention of HTLV-1 vertical transmission in Japan.
